# Role for RTX-family toxin HlyA of extraintestinal pathogenic *Escherichia coli* in serum resistance

**DOI:** 10.1093/femsmc/xtaf009

**Published:** 2025-07-02

**Authors:** Naoise McGarry, Stephen G J Smith

**Affiliations:** Department of Clinical Microbiology, School of Medicine, Trinity College Dublin, D08 RX0X, Dublin, Ireland; Department of Clinical Microbiology, School of Medicine, Trinity College Dublin, D08 RX0X, Dublin, Ireland

**Keywords:** *Escherichia coli*, LPS, haemolysin, HlyA, capsule, exotoxin, serum resistance, bloodstream infection, sepsis, ExPEC

## Abstract

Extraintestinal pathogenic *Escherichia coli* (ExPEC) is a major cause of urinary tract infections, bacteraemia, and sepsis. CFT073 is a prototypic, urosepsis isolate of sequence type (ST) 73. ST73 isolates are associated with higher virulence scores than other pandemic clonal groups, such as ST131. This laboratory, among others, has previously shown that strain CFT073 is serum-resistant, with virulence factors such as the exopolysaccharide capsule and other extracellular polysaccharides imparting resistance to the complement system. In this study, it was shown that culture supernatants were protective in standardized serum killing assays, when compared to cultures standardized in fresh medium. Diluting cultures in fresh medium in place of conditioned medium significantly increased sensitivity of CFT073 to serum, indicating that a secreted factor may provide resistance to serum. Haemolysin, a pore-forming toxin, is secreted by CFT073 in a calcium-dependent manner. This study found that a CFT073 *hlyA* mutant is significantly more sensitive to 50% serum than the wild-type, implicating haemolysin in the response of CFT073 to serum. In addition to acting as a toxin upon secretion, it has been shown previously that HlyA forms a complex with lipopolysaccharide (LPS), which permits modulation of host immune responses by HlyA whilst cell-associated. The effect of HlyA on capsule expression and serum resistance was examined and characterized in this study, with results indicating that perhaps the HlyA–LPS complex interacts with surface capsule. This study is the first to identify haemolysin as a virulence factor promoting resistance to serum in CFT073, acting whilst associated with the cell.

## Teaser

A common exotoxin of extraintestinal pathogenic *Escherichia coli* (ExPEC) was shown in this study to contribute to serum resistance, potentially through interactions with surface capsule and other surface-associated factors in an ExPEC prototype, CFT073.

## Introduction

Extraintestinal pathogenic *Escherichia coli* (ExPEC) is a collective term for pathogenic strains of *Escherichia coli*, which are responsible for causing infections outside of the gastrointestinal tract (Dale and Woodford 2015). Infections caused by ExPEC include urinary tract infections (UTI), neonatal meningitis, and bloodstream infections (Fratamico et al. 2016). These strains possess genes encoding specialized virulence factors, which enable the bacteria to colonize the extraintestinal sites, in addition to evasion of the host immune responses (Miajlovic and Smith 2014). Bloodstream infections can subsequently result in sepsis, fuelled by an disproportionate inflammatory response to the presence of bacteria and/or endotoxin in the bloodstream (Croxen and Finlay 2010).

Human serum consists of a variety of highly effective innate immune responses to *E. coli* colonization and bloodstream infection, including the complement system and lysozyme (Miajlovic and Smith 2014). Thus, in order for ExPEC to successfully colonize the host and survive in the blood, these bacteria encode several virulence factors, which confer serum resistance. Extracellular polysaccharide factors such as colanic acid, the LPS component O antigen, and the surface-associated polysaccharide capsule (K capsule) comprise the extracellular glycome and have been implicated in the resistance of ExPEC to human serum (Buckles et al. 2009, Miajlovic and Smith 2014, Miajlovic et al. 2014, Sarkar et al. 2014). A review previously published by this group explores the bactericidal components of human serum, and how ExPEC overcome these defenses, in detail (Miajlovic and Smith 2014). Additionally, a recent research publication by our group explored the total contribution of O antigen and capsule to serum resistance in an ExPEC prototype strain, CFT073 (serotype O6: K2: H1). The aforementioned study showed that double mutant lacking both capsule and O antigen was almost, but not entirely sensitive to serum, implicating additional factors in serum resistance (McGarry et al. 2024).

Exotoxins have been shown to play an important role in ExPEC pathogenesis, particularly in the early stages of infection and dissemination, often permitting access to deeper tissue as well as host cell invasion (Subashchandrabose and Mobley 2015). Additionally, toxins commonly expressed by ExPEC can accelerate and increase host damage and the inflammatory response (Kaper et al. 2004). The most common exotoxins encoded by ExPEC include *hlyC*-, *hlyA hlyB-*, and *hlyD*-encoded α-haemolysin, *cnf1* (cytotoxic necrotizing factor 1), *sat* (secreted autotransporter toxin), *pic* (protease involved in colonization), and *vat* (vacuolating autotransporter protein) (Logue et al. 2012, Miajlovic et al. 2016).

Haemolysin is a RTX exotoxin family, 107 kDa toxin, which is encoded and transported by products of the *hlyCABD* operon (Herlax et al. 2005). *hlyC* encodes a protein required for the posttranscriptional acetylation and activity of the toxin, *hlyA* encodes the nascent polypeptide, which is secreted by HlyBD (*hlyBD*-encoded) in a sec-independent manner following HlyC acetylation. Mature HlyA (or haemolysin) is similar to other RTX family toxins in its binding to Ca^2+^ through a conserved nine amino acid sequence, an interaction essential for HlyA activity (Rowe et al. 1994). Only around 15% of commensal *E. coli* isolates encode *hlyCABD*, in contrast to ~31%–48% of cystitis isolates (Bert et al. 2011). Interestingly, the percentage of isolates encoding *hlyCABD* is higher again in isolates of more severe UTIs, with 78% of pyelonephritis and bacteraemia isolates encoding HlyA (Ristow and Welch 2016). These findings indicate that HlyA is correlated with ExPEC infections, in perhaps contributing to disease severity. In fact, some ExPEC isolates, such as *E. coli* 536, possess more than one *hlyCABD* operon. Strain 536 possesses two intact copies of the haemolysin operon, located on distinct pathogenicity islands and playing roles in 536 virulence at distinct stages of growth (Nagy et al. 2006). The role of haemolysin in ExPEC pathogenesis is multifaceted and appears to vary depending on the *E. coli* isolate, the host cell upon which the toxin is acting, as well as site of infection. Several studies conducted in CFT073 backgrounds have shown that HlyA has a complex role in pathogenesis, displaying cytotoxic as well as immunomodulatory properties (Wang et al. 2020).

In addition to being secreted as an exotoxin, HlyA can also remain cell-associated through interactions with LPS. HlyA is an amphipathic molecule and associates with LPS through hydrophobic and electrostatic interactions between positively charged amino acids exposed on the surface of HlyA and the negatively charged phosphate groups of LPS (Herlax et al. 2005, Ristow and Welch 2016). In addition, due to its net negative charge, LPS serves as a Ca^2+^ reservoir when the toxin is cell-associated, with calcium being essential for HlyA toxicity (Bielaszewska et al. 2014). Ca^2+^ ions bind to the repeats within the HlyA protein and increase the affinity of HlyA for LPS (Herlax et al. 2005). The nature of LPS–HlyA activity is largely unexplored, however LPS is assumed to prevent the aggregation and degradation of HlyA, as well as to promote its activity (Czuprynski and Welch 1995). Reinforcing the importance of the relationship between LPS and HlyA, was data from Baeur and Welch, which showed that haemolysin transcription was downregulated greater than 2-fold in *waaC* mutants, resulting in significantly less toxin secreted (Bauer and Welch 1997). Mutants deficient in *waaG, waaF*, and *rfaE* were also shown to display significant reductions in haemolytic activity and *hlyCABD* transcription relative to bacteria with wild-type core polysaccharide (Nhu et al. 2019). Moreover, the HlyA-containing supernatants from core polysaccharide mutants were significantly less potent in their activity relative to the HlyA secreted from a smooth LPS background in the wild-type (Bauer and Welch 1997). This phenomenon wherein rough *E. coli* backgrounds produce HlyA with reduced activity compared to that produced from smooth backgrounds has been observed in several other strains and isolates (Bauer and Welch 1997, Herlax et al. 2005). Such findings indicate that the relationship between LPS and HlyA is important in ExPEC virulence, with the genes encoding both factors being implicated in a regulatory feedback loop, despite being under the control of distinct promoters (Ristow and Welch 2016, Liu et al. 2020).

The specific role, if any, played by HlyA in the bloodstream infection stage of ExPEC pathogenesis remains unclear. For example, strain 536 (O6: K15: H31) displayed attenuation in a murine bloodstream infection model upon mutation of its two *hlyCABD* operons (Brzuszkiewicz et al. 2006). Additionally, in a zebrafish bloodstream infection model conducted with ExPEC strain UTI89, *hlyA* mutants were significantly attenuated, proving nonlethal in comparison to 80% lethality in the wild-type within the first 24 h (Ristow and Welch 2016). Interestingly, infections caused by *E. coli* isolates deficient in HlyA and capsule were found to have a negative predictive value for sepsis in infant boys with UTI, thus indicating roles for capsule and HlyA in developing disseminated infections and/or resistance to serum (Bonacorsi et al. 2006). Similarly, HlyA status was correlated with mouse lethality in murine subcutaneous infection models of infection, as well as with higher levels of serum resistance compared to HlyA-negative isolates (Johnson et al. 2006, Crémet et al. 2016). Moreover, in a study examining 91 smooth strains of EXPEC and their respective sensitivity to serum, production of HlyA was correlated with O type (namely O4, O6, O18, and O75), as well as relatively higher levels of serum resistance compared to those without haemolysin (Hughes et al. 1982). HlyA has been shown to be implicated in dissemination and causing fulminant sepsis in a mouse infection model, although it was not explored whether HlyA was essential for bloodstream survival or simply promoted ExPEC ascension of the urinary tract and subsequent dissemination, thus resulting in sepsis (Johnsen et al. 2019). In fact, none of the aforementioned studies determined whether HlyA itself was implicated in serum resistance or bloodstream survival, or if the toxin increased morbidity and mortality through accelerating disease progression. Whilst the aforementioned studies highlight a key role for HlyA in ExPEC dissemination, it is important to explore whether HlyA plays a role in serum resistance, particularly given the importance of interactions with LPS, a known serum resistance factor (Grozdanov et al. 2002, Sarkar et al. 2014, McGarry et al. 2024). Thus, the role of HlyA in ExPEC prototype CFT073 serum resistance was the focus of this study.

## Methods

### Bacterial strains and culture conditions

The bacterial strains and plasmids utilized in this study are listed in Tables S1 and S2. Bacteria were grown overnight (~18 h) in LB Lennox/Luria broth (LB) NaCl, 5 g/l, tryptone, 10 g/l yeast extract, 5 g/l or on LB agar (Sigma) at 37°C. Bacteria harboring temperature sensitive plasmids pCP20 and pKD46 were cultured at 30°C. Broth cultures were shaken at 150 rpm for routine overnight culturing. Where required, antibiotics were added to growth media at the following concentrations: 10 μg/ml, gentamicin; 50 μg/ml, kanamycin; 100 μg/ml, and carbenicillin (all Sigma).

### Mutagenesis

Mutants were constructed as per the Lambda (λ)-Red recombination protocol as detailed by Datsenko and Wanner (2000). Kanamycin (source; pKD4) and gentamicin (pMH2) resistance genes were amplified through polymerase chain reaction (PCR) by primers, which had been designed to contain homologous flanking sequences to the target genes. Amplicons were purified using the Monarch DNA and PCR Cleanup Kit (New England Biolabs) and precipitated as per the Co-Precipitant Pink (Bioline) protocol before resuspension in 4 μl molecular-grade water (Thermo Fisher). The λ-red recombination genes on the pKD46 vector were induced through the addition of l-arabinose (final concentration 10 mM, Sigma) to CFT073/pKD46 cultures for 1.5 h at 30°C. The temperature-sensitive pKD46 plasmid was removed from CFT073 through incubation at 37°C. Putative mutants were confirmed through PCR. All mutant strains are listed in Table S1. Plasmids vectors used to amplify antibiotic resistance cassettes for mutagenesis are listed in Table S2. All oligonucleotides used for recombination and mutant screens are listed in Table S3. To remove antibiotic resistance cassettes, mutants were transformed with pCP20, which possesses the yeast flippase gene FLP, resulting in a marker-less mutant. The protocol followed for FLP-mediated cassette removal is detailed in (Datsenko and Wanner 2000).

### Haemolysis assays

To visualize haemolytic colonies on blood agar plates, colonies of CFT073, DH5 alpha and derivatives were streaked onto Columbia agar supplemented with 5% Defibrinated Horse Blood (E&O laboratories, Ireland). To quantify haemolysis, a 5% red blood cell (RBC) solution was prepared in phosphate-buffered saline (PBS) and 10 mM CaCl_2_, before 100 ul serial-dilutions of culture supernatant from CFT073, DH5 alpha and derivatives were mixed with 100 ul of the RBC solution. The blood/supernatant mixtures were incubated in a Greiner 96-well plate for 1 h at 37°C. LB was used in place of culture supernatant as a negative control, and 1% Triton X-100 was used as a positive control. After incubation, samples were centrifuged, and relative haemoglobin release was calculated after measuring the optical density at 540 nm (OD_540_). Percent haemolysis was calculated by setting the positive control as 100% haemolysis.

### RNA extraction and RT qPCR

Approximately 1 × 10^7^ bacterial cells were centrifuged at 12 000 × *g* for 1 min before beginning RNA extraction as per the Monarch Total RNA Miniprep Kit protocol (New England Biolabs). RNA was eluted in 30 μl nuclease-free water (Millipore) and quality and concentration were verified by Qubit and Nanodrop analysis. Nanodrop 260/280 and 260/230 scores were used to ensure RNA purity and lack of contamination by proteins or DNA. Real-time quantitative PCR (RT-qPCR) was used to quantify gene expression in *E coli*. The Luna® Universal One-Step RT-qPCR Kit was used for all RT-qPCR reactions in 20 µl volumes with 10 ng RNA used as template. Standard curves were generated with fix 2-fold serial dilutions of wild-type CFT073 RNA. RT-qPCR reactions were set up in triplicate in MicroAmp Fast Optical 96-well reaction plates (Applied Biosystems) and run on the StepOnePlus Real-Time PCR System (Thermo Fisher). Default “Fast” RT-qPCR and melt curve settings were utilized. All oligonucleotides used for RT-qPCR are listed in Table S3. Data analysis was carried out using the StepOne software or Prism GraphPad. Housekeeping gene *rplT* was used as an internal control. Relative expression (or “RQ”) is calculated automatically by the instrument. RQ is the fold change compared to the calibrator (calibrator is usually the untreated or wild-type sample—the calibrator is indicated in each figure legend). The calibrator has a RQ value of 1 and the values for the other samples are the fold-change relative to the calibrator.

### SDS-PAGE

To prepare whole-cell lysates for SDS-PAGE, 1 ml overnight or exponential cultures of CFT073 strains were centrifuged at 13 000 × *g* and resuspended in 1x Laemelli (Sigma) to a final concentration of 10 OD_600nm_. Samples were boiled at 100°C for 10 min and allowed to cool before adding 20 μg/ml Proteinase K (Sigma) and incubating at 56°C for 1 h. 20 μl of samples were stored at −20°C or run on a 4%–20% TruPAGE Precast gel at 180 V in 1X TruPAGE SDS Buffer (both Sigma). Following electrophoresis, gels were stained according to the Pro-Q™ Emerald 300 Lipopolysaccharide Stain Kit (Thermo Fisher) and visualized under the Quantity One® (Bio-Rad) imaging system. Alternatively, gels were stained with 0.125% Alcian blue solution (Sigma) to observe total polysaccharide content.

### Western blot and densitometry

CFT073 whole cell lysates were separated by sodium dodecyl sulfate–polyacrylamide gel electrophoresis (SDS-PAGE) on TruPAGE precast gels as described above. Gels were transferred onto polyvinyl difluoride using the iBlot 2 (Thermo Fisher) for dry transfer. Membranes were blocked in 5% bovine serum albumin (BSA) (Sigma) in 1x PBS supplemented with 0.1% Tween 20 (0.1% PBS-T) for 1 h at room temperature (RT). Membranes were incubated in anti-O6 or anti-K2 primary antibody (Statens Serum Institut) diluted 1/500 in 5% BSA 0.1% PBS-T for 90 min at RT. Membranes were washed three times for 10 min in 0.1% PBS-T before incubating with antirabbit-HRP antibody (Cell Signalling Technologies) diluted 1/10 000 in 5% BSA 0.1% PBS-T for 45 min at RT. Membranes were washed four times before staining for 5 min with Pierce ECL Western Blotting Substrate (Thermo Fisher) as per the manufacturer’s protocol. Membranes were imaged under the ImageQuant Las4000 (GE Healthcare Life Sciences). ImageStudio Lite (v 5.5.4) was utilized to relatively (no absolute values) quantify bands from western blot .TIFF images. The quantitative values reflect the relative amount of polysaccharide as a ratio of each band relative to the gel background.

### ELISA

Cultures were washed twice in PBS and standardized to OD_600_ = 0.1 in PBS before plating 25 μl onto a poly-l lysine treated 96-well plate (Greiner) for overnight incubation at 4°C. The plates were centrifuged for 1 min at 10 000 × *g* before the removal of supernatant and addition of 100 μl 0.1% v/v glutaraldehyde (Sigma) for 10 min at RT. Wells were washed three times with PBS-T before the addition of 200 μl 5% (w/v) skimmed milk (Marvel) in PBS-T). Plates were blocked for an h at 37°C before the addition of anti-OmpF or anti-K2 antibody diluted 1/1000 to each well for incubation at 37°C for 1 h. Wells were washed three times vigorously with PBS-T before addition of 200 μl antirabbit-AP antibody (Cell Signalling Technologies) at 1/10 000 dilution to each well. Wells were washed for a final three times with PBS-T before adding 100 μl of the 1-Step PNPP (Thermo Fisher) to each well. The substrate was mixed thoroughly by gently agitating the plate at RT for 30 min. To stop the reaction, 50 μl 2 M NaOH was added. Absorbance was measured at 405 nm.

### Serum killing assay

The serum sensitivity of CFT073 and mutant derivatives was examined by exposing cells to 50% normal human serum (NHS) (Biowest) and heat-inactivated serum (HIS) for 90 min at 37°C and determining survival through viable counting as previously described (Miajlovic et al. 2014). Serum was heat-inactivated by heating at 56°C for 30 min. Percentage survival was calculated as a fraction of CFU/ml at T90 in NHS over CFU/ml at T90 in HIS (x100). Serum resistance was measured at logarithmic (OD_600nm_ = 0.6) and stationary phase (OD_600nm_ = 3.0). Standardized assays were conducted in order to directly compare response of stationary phase and logarithmic cells to serum at equivalent cell numbers. Stationary phase cultures (OD_600_ = 3.0) were diluted to OD_600_ = 0.6 in conditioned LB (the cell-free growth supernatant of the strain).

### Construction of plasmids to complement *hlyCABD* mutant

The pCL1920 low copy number plasmid was chosen as the vector for HlyA complementation. *hlyCABD* was amplified with primers, which contained flanking sequences for *XbaI* and *KpnI* digestion. The 8.6 kb XbaI-*hlyCABD*-KpnI amplicon was purified by precipitation, before the product was digested with *XbaI* and *KpnI* and ligated to *KpnI/XbaI* digested pCL1920. The ligation mixture was transformed into DH5α chemically competent cells (New England Biolabs). Putative clones were screened by blue/white selection on LB agar plates supplemented with 100 μg/ml spectinomycin/40 μg/ml X-Gal/0.1 mM IPTG plates (all Sigma). Plasmid orientation was confirmed by restriction digest and plasmid sequence was first confirmed by Sanger sequencing (Source Bioscience) by primer walking, then absolutely confirmed with long-read Nanopore sequencing by Plasmidsaurus (USA).

### Bioinformatic analyses

To analyse the prevalence of exotoxins in *E. coli* bloodstream isolates, Illumina fastq paired-end reads were downloaded from the European Nucleotide Archive under the project name PRJEB44839 (Pöntinen et al. 2024). The fastq files were de novo assembled using SPAdes v3.13.1 under default parameters. Genomes were then analysed for the presence of toxins using the Virulence Factor Database (VFDB) via SeqSphere v64. The same platform was used to assign sequence types to assemblies, as per the *E. coli* cgMLST scheme.

### Graphing

All graphing was completed using GraphPad Prism (v.9.5.0). Graphical figures were made using Biorender (Biorender, online graphing tool).

### Statistical analysis

All statistical analysis was carried out using the GraphPad Prism software (v.9.5.0) unless otherwise stated. Statistical analysis was performed exclusively on biological replicates, wherein experiments were conducted a minimum of three (*N* = 3) or four times (*N* = 4). In all data, a *P*-value of ≤ .05 is denoted *; a *P*-value of ≤ .01 is denoted **; a *P*-value of ≤ .001 is denoted ***; and a *P*-value of ≤ .0001 is denoted ^****^.

## Results

### Supernatant is protective against the bactericidal effects of serum

This group has previously examined the contribution of surface-associated virulence factors, such as O antigen and capsule, to ExPEC serum resistance (McGarry et al. 2024). To determine whether secreted/supernatant factors, such as proteins, toxins, polysaccharides, or quorum-sensing autoinducers, contributed to serum resistance, cultures were standardized in either conditioned LB growth medium (i.e. cell-free but contains any secreted factors), or in fresh LB. For all strains examined, there was a reduction in serum resistance when standardized in fresh LB medium before exposure to 50% NHS in comparison to those standardized in conditioned medium (see Table 1). For wild-type, Δ*waaL* and Δ*waaG*, there was a 10-fold reduction in survival in 50% NHS following standardization in fresh LB. Wild-type survival dropped from 100% to 10% (*P =* .001), Δ*waaL* survival in 50% NHS dropped from 0.2% to 0.02%, *P =* .049, and Δ*waaG* dropped from 0.1% to 0.01%, *P =* .036. Capsule mutants Δ*ksl* and Δ*ksl* Δ*waaL* also displayed reductions in serum survival when standardized in fresh LB in place of conditioned LB with a 6-fold (*P =* .046) and 3.3-fold (*P =* .2) reduction in survival, respectively. Interestingly, the capsule mutants did not display as pronounced of a reduction in survival when supernatant was removed in comparison to the other strains, which do express capsule.

**Table 1.** Supernatant is protective against the bactericidal activity of serum.

	% Survival in 50% NHS		
Strain	Supernatant	LB	Fold change
Wild-type	100	10	10**
Δ*ksl*	3	0.5	8*
Δ*waaL*	0.2	0.02	10*
Δ*ksl* Δ*waaL*	0.001	0.0003	3.3
Δ*waaG*	0.1	0.01	10*

Cultures were grown to to OD_600_ = 0.6–0.8 and standardized in conditioned media or fresh LB to 1 × 10^8^ before exposure to NHS or HIS. *; *P* ≤ .05, **; *P* ≤ .01, utilized to represent statistical significance relative to cultures standardized in supernatant. Statistical analysis conducted by two-way ANOVA and Tukey’s multiple comparisons. *N* = 4. Comparisons between strains are not shown in the above table.

### Prevalence of common exotoxins in ExPEC genomes

The findings depicted in Table 1 indicated that a secreted factor may be implicated in serum resistance of strain CFT073. To guide the identification of the secreted factor, the prevalence of known exotoxins in CFT073 and other representative ExPEC genomes was determined. A recent study sequenced several-hundred *E. coli* bloodstream isolates from the UK and Ireland, which had been collected by the BSAC between 2012 and 2017 in order to examine clone success and antimicrobial resistance trends (Pöntinen et al. 2024). The genome sequences of these bloodstream isolates collected by BSAC served as the aforementioned “representative genomes” in the context of our study. Thus, the raw sequencing files were downloaded, assembled, and analysed. The use of whole genome sequencing data in our study was important to look beyond the CFT073 genome for putative secreted factors, as the results of the study should be subsequently considered in a wider context and applied to other successful clonal groups of ExPEC. Of 629 genomes analysed, the majority of bloodstream infection isolates belonged to ST73 (115 or 18.2%), the sequence type of prototypic ExPEC strain CFT073. ST131 (107 or 17%), ST69 (56 or 8.9%), ST95 (53 or or 8.4%), and ST12 (5.4%) were the other most frequently occurring sequence types, respectively (see Table S6). The 629 assemblies were screened for known ExPEC virulence factors using the VFDB, in order to determine the prevalence of exotoxins HlyA, Sat, Pic, and Vat within the cohort. Sat was the most frequently occurring exotoxin encoded by ExPEC bloodstream isolates, with 320 or 50.8% of isolates possessing the *sat* gene. The next most prevalent exotoxin encoded by isolates was Vat (316 or 50.2%), followed by HlyA (206 or 32.7%), and lastly, Pic (135 or 21.4%). The distribution of exotoxins across the distinct sequence types was nonrandom, with significant associations between ST73 and *hlyA, pic, sat*, and *vat* status, compared to other sequence types (Table S6). Whilst *vat* and *sat* were more prevalent than *hlyA* in ST73 and the other clinically relevant STs identified among the bloodstream isolate cohort, HlyA was the focus of this study due to a lack of previous work exploring the role, if any, HlyA may play in serum resistance, despite the toxin being so prevalent among isolates.

### Mutant deficient in HlyA lacks haemolytic activity

In order to determine whether HlyA contributes to serum resistance in CFT073, a mutant deficient in the exotoxin was constructed. The *hlyA* gene of the *hlyCABD* was replaced with a kanamycin resistance gene through homologous recombination using the λ-Red recombination method (see Table S1). Supernatants from wild-type CFT073 and the *hlyA* mutant were also subject to SDS-PAGE and staining with Coomassie blue for protein visualization. The *hlyA* mutant lacked the 116 kDa band, which corresponds to HlyA, as shown in Fig. S4. Additionally, the phenotypic characteristics of the CFT073 *hlyA* mutant were examined through several quantitative and qualitative assays, also depicted in Fig. S4.

### HlyA mutant is significantly more sensitive to serum during exponential growth

**Figure 1.**
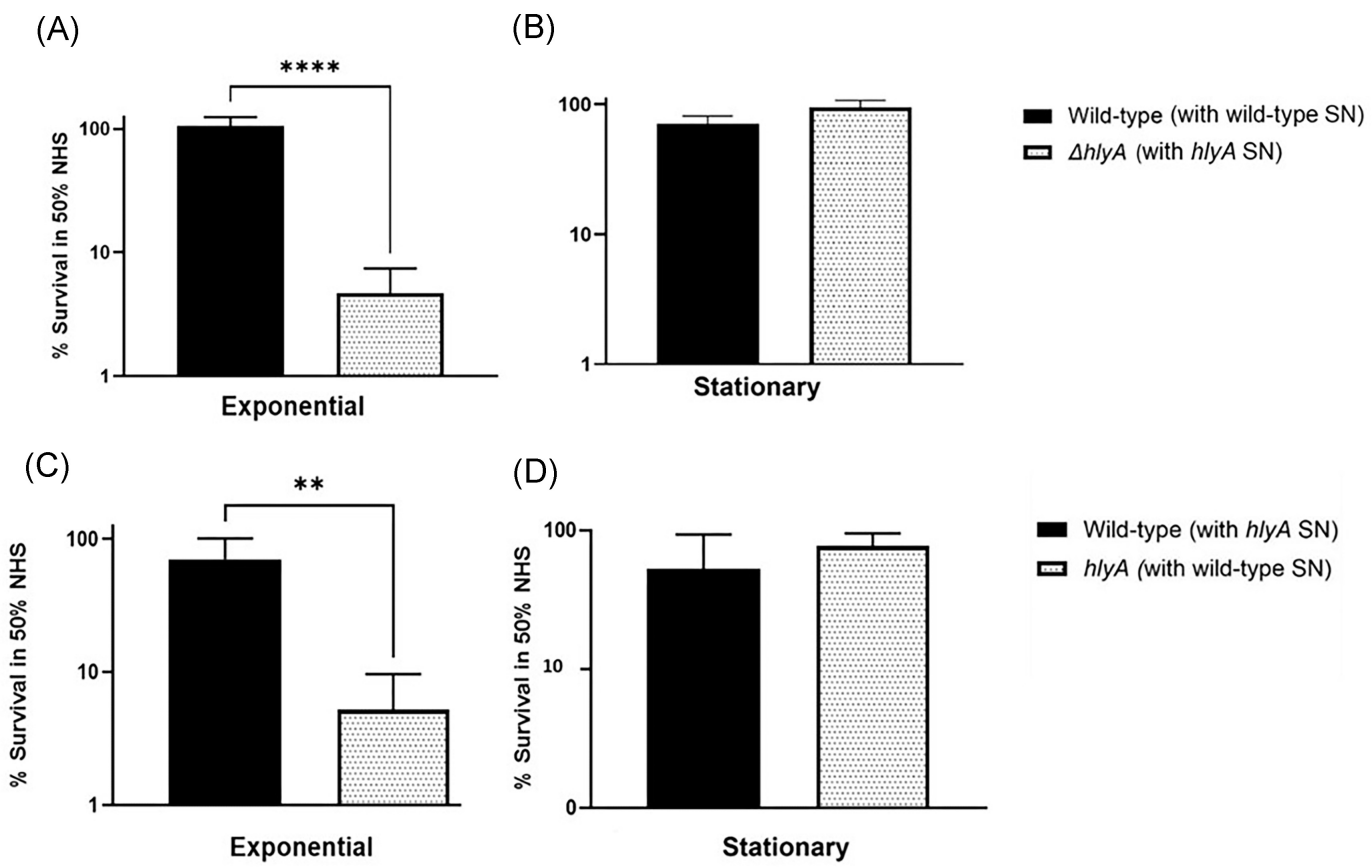
HlyA mutants are sensitive to serum during exponential growth. Cultures were grown to (A) exponential OD_600nm_ = 0.6 and (B) stationary phase OD_600nm_ = 3.0 (standardized to OD_600nm_ = 0.6) in conditioned LB medium before diluting 1:2 in NHS. For the supernatant swap assay, cultures were grown to (C) exponential and (D) stationary phase as before, prior to standardizing in the conditioned LB of the other strain (e.g. wild-type was standardized in HlyA-deficient supernatant and *vice versa*) before diluting 1:2 in NHS. Percentage survival was calculated as a fraction of CFU/ml at T90 in NHS over CFU/ml at T90 in HIS x 100. Statistical significance calculated by unpaired one-way ANOVA and shown relative to wild-type for each assay. *Y*-axis in Log 10. *N* = 4 biological replicates.

Cultures were subject to a serum killing assay to determine whether the HlyA mutant displayed attenuated survival in human serum. The percentage survival of exponential CFT073 strains after 90 min of exposure to 50% NHS is depicted in Fig. 1(A). These results showed that wild-type CFT073 was completely resistant to 50% NHS at exponential growth phase (102% survival). CFT073 Δ*hlyA* displayed a significant reduction in serum resistance (4.6% survival) in comparison to wild-type (*P* < .0001) during exponential growth. Conversely, stationary Δ*hlyA* displayed no significant difference in serum sensitivity in 50% NHS (96% survival) in comparison to wild-type (88%), *P =* .0637 (Fig. 1B). In order to determine whether the increased sensitivity of Δ*hlyA* to 50% NHS during exponential phase was due to the secretion of HlyA, the supernatant of wild-type was utilized to standardize exponential Δ*hlyA* cultures prior to exposure to 50% NHS. There was no increase in the serum sensitivity of *hlyA* mutants upon the addition of wild-type supernatant (Fig. 1C). Similarly, the addition of HlyA-lacking supernatants to wild-type cultures prior to the addition of 50% NHS did not significantly decrease the percentage survival of wild-type, compared to wild-type cultures standardized in their own HlyA-containing supernatant (Fig. 1D). These data indicate that secreted HlyA does not directly interact with complement proteins in an *in vitro* serum killing assay and there are likely other secreted/supernatant factors contributing to serum resistance.

### *hlyA* mutants possess reduced capsule relative to wild-type

**Figure 2.**
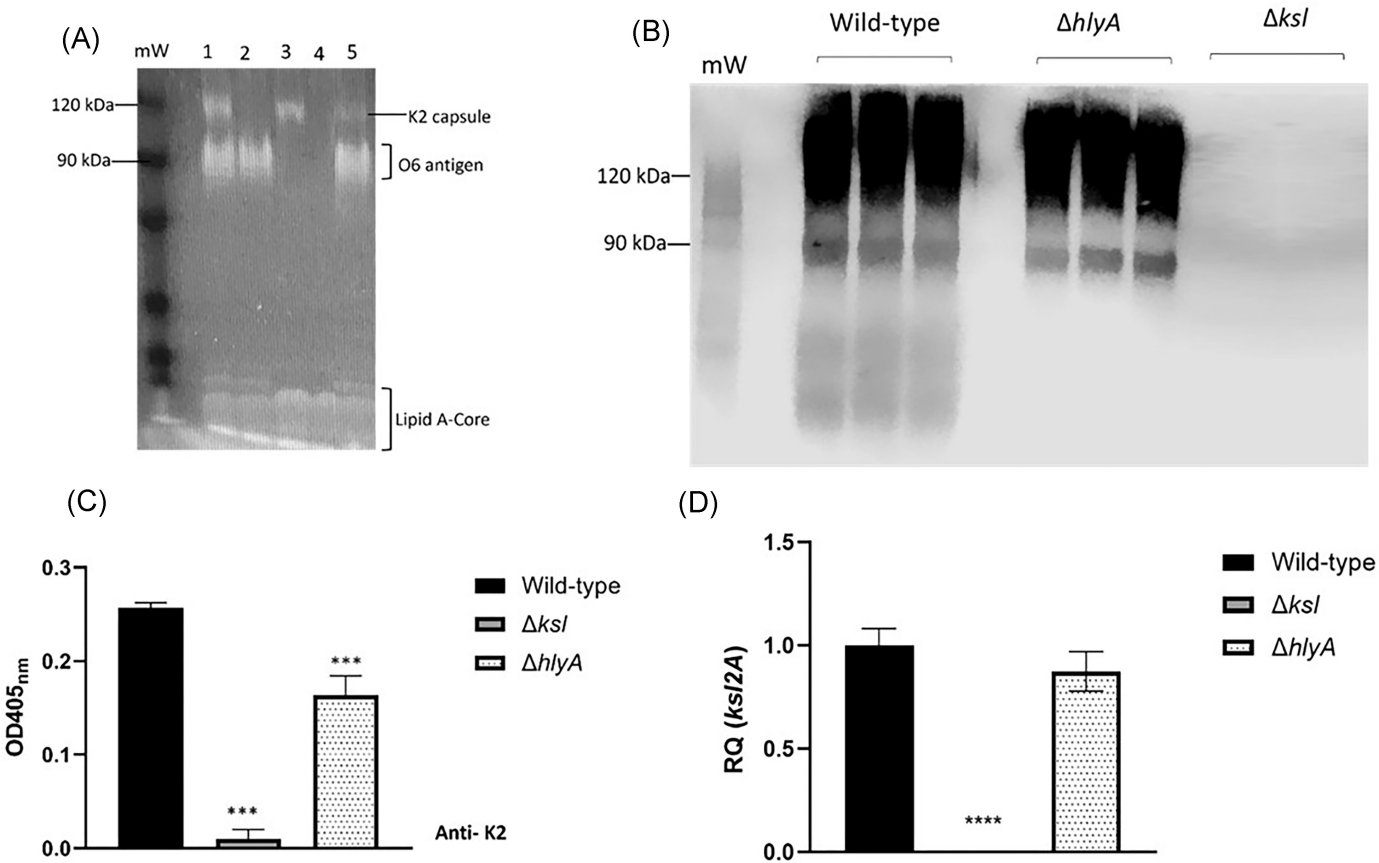
HlyA mutants display posttranscriptional changes to capsule. Cultures were grown to exponential OD_600nm_ = 0.6 and whole cell lysates were separated by SDS-PAGE and subject to LPS straining (A; *N* = 3, *N* =1 shown). Lane 1; wild-type, 2; Δ*ksl*, 3; Δ*waaL*, 4; Δ*ksl* Δ*waaL*, and 5; Δ*hlyA*. Whole cell lysates were also subject to western blot (B; *N* = 3). Additionally, exponential cells were subject to ELISA (C; *N* = 4) and RT-qPCR (D; *N* = 4) to examine detectable K2 capsule and capsule gene expression, respectively. Statistical significance calculated by one-way ANOVA and shown relative to wild-type. Anti-K2 = anti-K2 antibody used in ELISA.

As discussed above, the role of haemolysin in CFT073 serum resistance was shown to be unrelated to HlyA secretion or activity of the free toxin in supernatants. To ascertain whether the reduced serum resistance seen in *hlyA* mutants was due to changes in the glycome composition, SDS-PAGE (Fig. 2A), western blot (Fig. 2B), enzyme-linked immunosorbent assay (ELISA) (Fig. 2C), and RT-qPCR (Fig. 2D) analyses of K2 capsule were conducted to compare wild-type and Δ*hlyA* capsule phenotype and expression. ELISA quantification of capsule showed that there was a significant reduction in detectable K2 capsule relative to wild-type (*P =* .0003). Similarly, there was qualitatively less K2 capsule visible on a stained SDS-PAGE gel and western blot. Interestingly, the most obvious difference in capsule between wild-type and Δ*hlyA* as visualized by western blot, is at a lower molecular weight than the typical bands seen at 90 and 120 kDa, although these bands also depicted slightly more capsule in wild-type lysates compared to the HlyA-deficient mutant lysates. Next, RT-qPCR was conducted on wild-type, *ksl* and *hlyA* mutants to compare *ksl2A* transcript levels in order to determine whether *hlyA* mutants display changes to capsule gene expression due to a potential feedback loop via RfaH, as seen with LPS mutants in (Nhu et al. 2019). There was no significant change to capsule gene expression in the *hlyA* mutant, compared to wild-type, which infers that the change to capsule seen in Fig. 2(B) and (C) are posttranscriptional and due to changes to the cell surface, rather than transcriptional changes.

### Complementation of HlyA restores haemolysis and serum resistance

**Figure 3.**
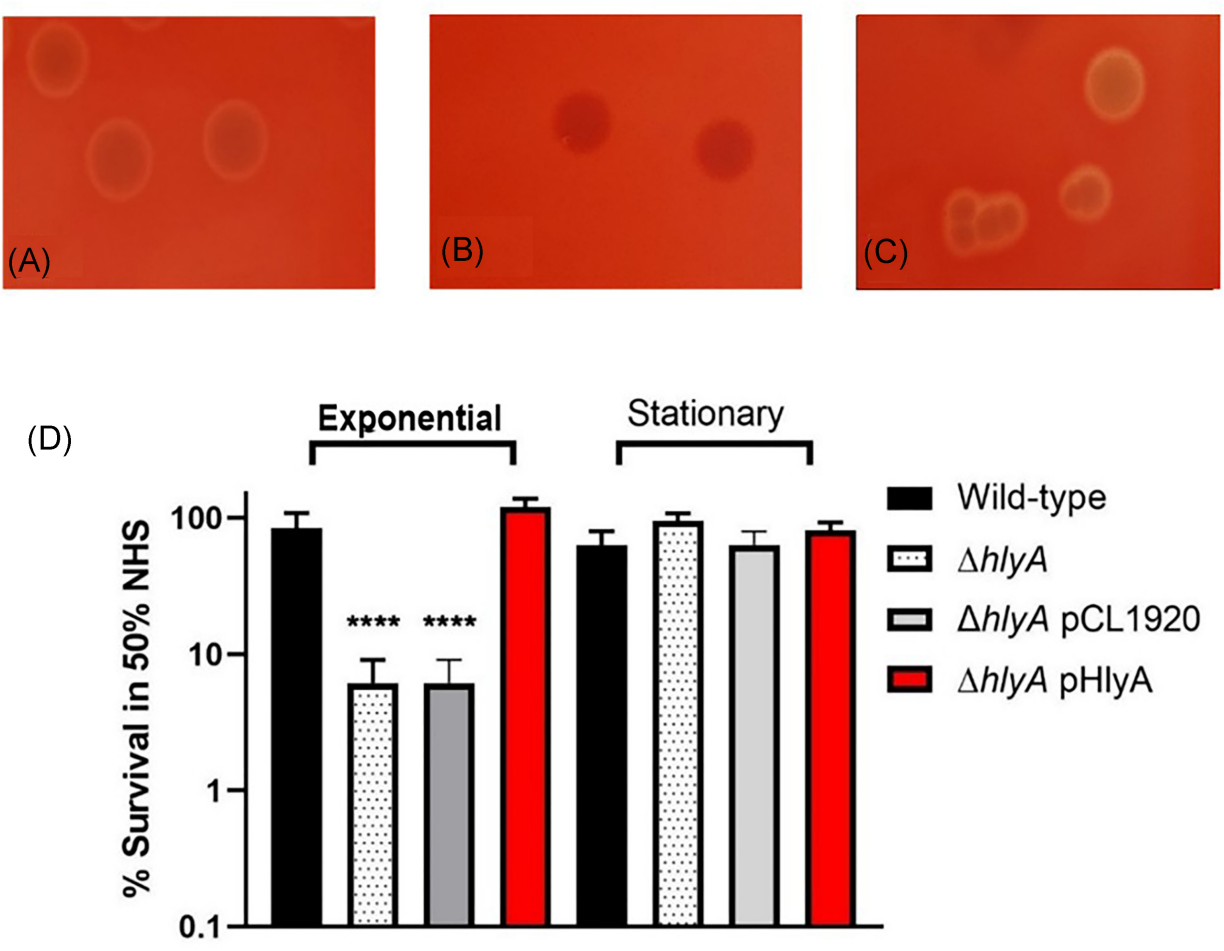
Complementation of *hlyA* restores haemolysis and serum resistance. (A) Wild-type colonies are haemolytic, (B) Δ*hlyA* colonies are not haemolytic on blood agar, and (C) complemented mutants display a restoration of haemolysis, to levels comparable to wild-type. (D) Survival of wild-type, Δ*hlyA*, and Δ*hlyA* pHlyCABD at exponential and stationary phase upon exposure to 50% NHS. Strains were grown to OD_600nm_ = 0.6 (exponential) or OD6_00nm_ = 5.0 and incubated in 50% HIS and NHS for 90 min at 37°C. Statistical significance determined by One-way ANOVA and Dunnet’s multiple comparisons test. Significance shown relative to wild-type. *n* = 4. ^****^; *P* < .0001. *N* = 4 biological replicates.

To confirm the contribution of HlyA in serum resistance of CFT073, a plasmid vector containing the *hlyCABD* operon was constructed (see Fig. S5). Cultures were streaked onto blood agar plates to observe for haemolytic activity. Haemolysis was observed on the wild-type and complemented mutant agar plates, but not on Δ*hlyA* plates, as shown in Fig. 3(A)–(C). These data show that the vector pHlyCABD successfully restores the expression, transport, and secretion of HlyA. Lastly, as depicted in Fig. 3(D), during exponential and stationary phase, Δ*hlyA* pHlyCABD displayed no significant difference in resistance to serum compared with wild-type, indicating that restoration of HlyA expression restores resistance to serum. These data confirm the contribution of HlyA to serum resistance of CFT073.

### Mutations to LPS core genes prevents haemolytic activity

**Figure 4.**
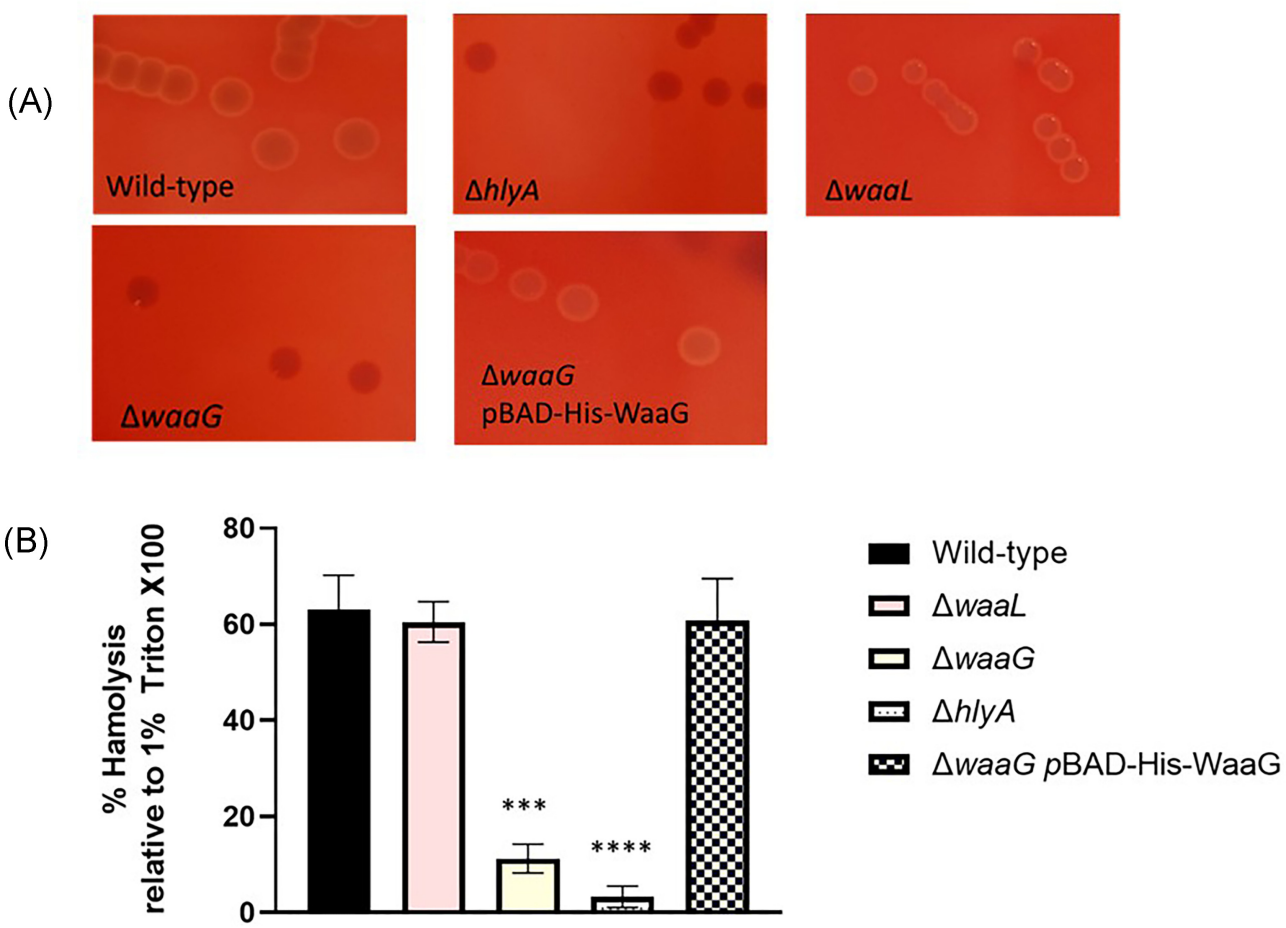
Feedback loop exists between *waa* genes and the *hly* operon. (A) CFT073, mutant derivatives and complemented *waaG* mutant were plated on blood agar plates to observe haemolytic activity (if any). These results were mirrored by quantitative haemolysis assays performed in (B), as before, wherein 1% TritonX100 served as a positive control for haemolysis and uninoculated LB broth served as a negative control. Statistical significance determined by One-way ANOVA and Dunnett’s multiple comparisons test. Significance shown relative to wild-type. *n* = 4. ***; *P* < .001, ^****^; *P* < .0001. *N* = 4 biological replicates

As discussed previously, it has been shown that mutations to genes involved in LPS core biosynthesis result in decreased *hlyCABD* expression in several strains of *E. coli*, such as CL632 and several strains belonging to the ST131 clonal group (Bauer and Welch 1997, Nhu et al. 2019, Pagnout et al. 2019). However, as this phenomenon had not previously been explored in an ST73 genetic background, mutant derivatives of CFT073 lacking the gene *waaG* (outer core glycosyltransferase), and *waaL* (O antigen ligase) were subject to haemolysis assays and compared to wild-type. As depicted in Fig. 4(A), wild-type displayed visible haemolysis and the *hlyA* mutant was nonhaemolytic, with these results quantifiably mirrored in Fig. 4(B). Additionally, the *waaG* mutant displayed no visible or quantifiable haemolysis, correlating with findings from other groups about coregulation of *waa* genes and *hlyCABD* (Bauer and Welch 1997). Interestingly, the CFT073 *waaL* mutant displayed haemolysis on blood plates at levels, which appeared comparable to wild-type, which indicate the transcriptional feedback loop between *waa* genes and the *hly* operon does not include the *waaL* gene in CFT073, which is under a distinct promoter from the other *waa* genes. Complementation of the *waaG* mutant using the pBAD-His-WaaG vector from (Muheim et al. 2016) restored haemolytic activity in CFT073, demonstrating that the *waaG* mutation was nonpolar.

### pHlyA renders DH5α haemolytic, but does not confer resistance to serum

**Figure 5.**
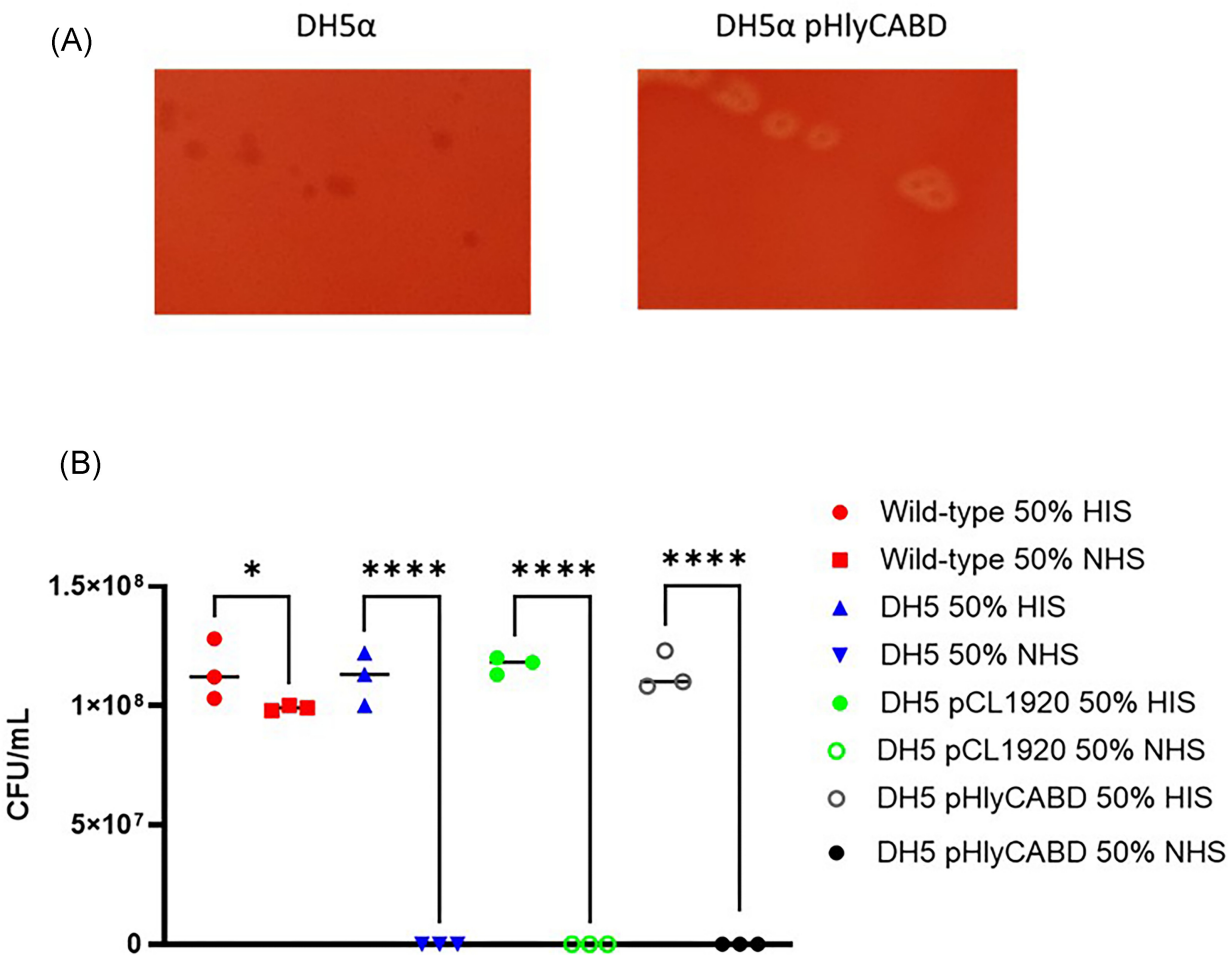
HlyA does not confer resistance to nonpathogenic strain *in vitro*. (A) DH5α colonies are nonhaemolytic, whilst DH5α pHlyCABD are haemolytic. (B) Exponential (and stationary phase—not shown) cultures were standardized to 1 × 10^8^, exposed to 50% NHS and HIS for 90 min at 37°C prior to plating, overnight incubation, and CFU/ml counting. Wild-type = CFT073. Statistical significance determined by One-way ANOVA and multiple comparisons. ^****^ = *P* < .0001. *N* = 4 biological replicates.

Some K-12 strains such as DH5α are nonpathogenic and do not typically express HlyA. Additionally, DH5α serves as a control for serum sensitivity throughout this study, with cultures displaying 0% survival in 50% NHS. To determine whether pHlyCABD would impart serum resistance to DH5α, chemically competent DH5α were transformed with the vector (in addition to the empty vector control, pCL1920) and bacteria were plated on blood agar plates to observe for haemolysis. As seen in Fig. 5(A), DH5α is typically nonhaemolytic, and no haemolysis is observed surrounding wild-type colonies. However, upon the addition of pHlyCABD, DH5α colonies possess haemolytic activity. Despite pHlyCABD rendering DH5α haemolytic, the expression of the *hlyCABD* operon did not confer serum resistance to the strain (depicted in Fig. 5B). DH5α is completely sensitive to serum with and without the addition of the pHlyCABD vector, with 0% survival after 90 min incubation at 37°C. Figure 5(B) depicts a serum killing assay conducted when cells were grown to exponential phase. A stationary phase assay was also conducted, but not shown as results were comparable. Finally, the experiments included empty vector controls of DH5α and CFT073 expressing pCL1920, with results showing that pCL1920 does not confer or attenuate serum resistance with regards either strain. Together, these data indicate that, as hypothesized, the secretion of HlyA is not how the toxin contributes to serum resistance but instead through association with LPS and the R1 core resulting in maximum capsule retention, all of which are molecules K-12 is devoid of.

## Discussion

This study demonstrated that strain CFT073 supernatant is protective against serum. For all CFT073 derivative strains examined, there was a reduction in serum resistance when standardized in fresh LB medium before exposure to 50% NHS in comparison to those standardized in conditioned medium. For wild-type, Δ*waaL* and Δ*waaG* there was a 10-fold reduction in survival in 50% NHS following standardization in fresh LB. Capsule mutants Δ*ksl* and Δ*ksl* Δ*waaL* also displayed reductions in serum survival when standardized in fresh LB in place of conditioned LB with a 6-fold and 3.3-fold reduction in survival, respectively. Interestingly, the capsule mutants did not display as pronounced of a reduction in survival when supernatant was removed in comparison to the other strains, which do express capsule. These results indicate perhaps capsule is involved in serum resistance if released into supernatant. Intriguingly, capsule has previously been shown to be present in CFT073 supernatants, as discussed in Valle et al. (2006), McGarry et al. (2024). However, due to the reduction in survival seen across all strains when supernatant is removed, capsule is not the only secreted/supernatant factor which is protective against serum.

This study also found that secreted HlyA does not contribute to serum resistance of CFT073, as standardizing wild-type cultures in HlyA-deficient supernatant did not affect serum resistance. Thus, some of the other secreted exotoxins encoded by CFT073 may contribute to the protective effects of ExPEC supernatant upon exposure to serum. For example, the serine protease, Pic, is encoded by CFT073 (gene c0350, accession number: AE014075.1). Pic has been implicated in complement resistance, as the protease was shown to inhibit the activity of the classical complement pathway *in vitro* through cleavage of C3, C3b, C2, and C4 (Henderson et al. 1999, Abreu et al. 2015). In fact, it has been shown that a 30-min pretreatment of serum with Pic permitted the survival of K-12 nonpathogenic strain DH5α. Similarly, Sat, another secreted autotransporter protein, has been shown to cleave all complement proteins bar C1q and as with Pic, pretreatment of serum with Sat abrogated serum killing of K-12 strain DH5α (Freire et al. 2022). However, the contribution of Sat or Pic has not been explored in a CFT073 background, with the aforementioned studies primarily focusing on cloning the toxins into nonpathogenic strains to examine their effects.

Interestingly, we have shown that of 629 *E. coli* bloodstream isolates, over 50% genomes encoded Sat, with the toxin being prevalent amongst the most common and significant ExPEC clonal groups, such as ST131, ST73, and ST69. Additionally, the gene encoding Pic was present in 21.5% of bloodstream isolates, although this exotoxin is almost exclusively encoded by ST73 isolates, the most prevalent clonal group in the BSAC collection and also the clonal group associated with the highest virulence scores (Miajlovic et al. 2016, Pöntinen et al. 2024). Taken together, these findings highlight the prevalence of complement-degrading exotoxins in UK and Irish ExPEC bloodstream isolates, indicating that these exotoxins could be included or explored in toxoid vaccine development strategies. Future work by this group will include studies to determine the role (if any) for Sat and Pic in CFT073 serum resistance.

In this study, HlyA has been shown to play a role in CFT073 serum resistance, although not in secreted form. In previous work published by this lab, a dependency of K2 capsule on polysaccharides such as the lipopolysaccharide outer core and O6 antigen was shown (McGarry et al. 2024). The dependency of K2 on LPS highlights the intricate interactions, which occur at the surface of the cell envelope which contribute to cell integrity and composition/structure of the glycome. Interestingly, the effects of the *hlyA* mutation on serum resistance was only seen in cells grown to exponential phase and not in stationary phase cells. Similarly, changes to capsule were only seen in exponential cells. The growth phase-dependent regulation of the capsule has been explored previously, with different nucleoid associated proteins playing a role in the regulation of group 2 capsule gene expression (Aldawood and Roberts 2022). It was thought that perhaps the *hlyA* mutation resulted in changes to *ksl* expression directly or indirectly, through involvement in a feedback loop potentially involving RfaH, in a similar manner to the requirement of core gene expression for *hlyCABD* transcription (Nhu et al. 2019). This possibility of indirect cross-talk between *hlyCABD* and capsule genes would explain the difference in serum resistance between the *hlyA* mutant at exponential and stationary phase. It was also thought that if the reduction in capsule seen in *hlyA* mutants occurred on a transcriptional level, perhaps this was due to a feedback loop involving a regulator or NAP involved only in exponential phase capsule regulation such as IHF (Aldawood and Roberts 2022). RT-qPCR analysis of *ksl* gene expression showed no change to LPS (Fig. 2A) or capsule expression (Fig. 2D), indicating that the feedback loop (wherein core gene expression is required for haemolysin expression) is unidirectional, with LPS biosynthesis occurring despite the lack of haemolysin expression. Moreover, the changes to capsule do not occur by virtue of transcriptional changes at the capsule operon, indicating that the effect of HlyA on capsule levels (and serum resistance) occurs posttranscriptionally.

In this hypothetical model, HlyA may alter the charge of LPS/the outer envelope due to its known association with LPS O antigen and the core, which serve as reservoirs of Ca^2+^, essential for HlyA activity (Wang et al. 2020, Kolenda et al. 2021). The reduced capsule expression seen in *hlyA* mutants indicates that it may be the contribution of LPS–HlyA to cell integrity and capsule association which confers serum resistance. The role of HlyA–LPS in cell integrity and glycome composition correlates with the data which highlighted that pHlyCABD did not confer any serum resistance to DH5α in 50% NHS. Additionally, HlyA-containing supernatant did not enhance serum resistance of exponential Δ*hlyA*, and the HlyA-deficient supernatant did not reduce the resistance of wild-type to serum. Taken together, these two findings support the hypothesis that secreted HlyA does not have direct activity against complement or other antimicrobial components of serum and instead, through association with LPS, prevents complement deposition and killing through enhancing capsule retention at the cell surface.

**Figure 6.**
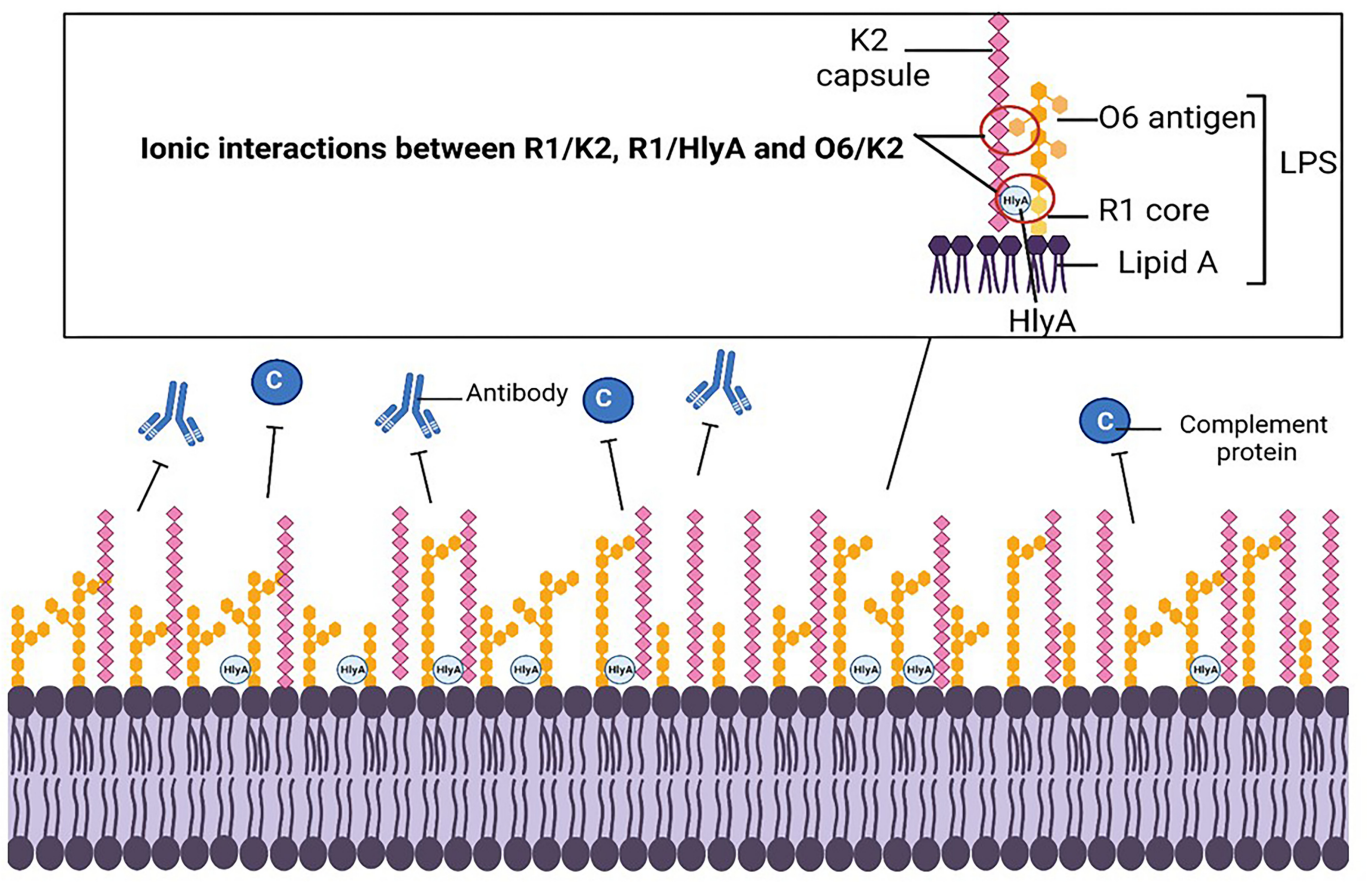
Hypothetical model for the role of HlyA in serum resistance. Graphical representation of the CFT073 outer envelope, including LPS and capsule. This study has shown that the R1 core and O6 antigen contribute to K2 capsule retention, likely through ionic interactions as detailed in other species such as *Klebsiella* (Fresno et al. 2006, Singh et al. 2022), as well as *E. coli* K1 (Jiménez et al. 2012). HlyA also associates with LPS (likely the core) through ionic interactions and as a result of findings in this study, is hypothesized to associate with K2 in a similar manner.

Interestingly, despite evidence that HlyA binds to smooth LPS, and that supernatants from cultures of smooth LPS phenotype are significantly more haemolytic than supernatants from rough strains, the CFT073 *waaL* mutant displayed haemolysis on blood plates at levels, which appeared comparable to wild-type. Thus, *waaL* is not included in the cross-talk, which occurs with respect to *waa* and *hlyCABD* expression. As mentioned, LPS from rough backgrounds, although less potent compared to supernatants from smooth backgrounds, still displayed toxicity and resulted in erythrocyte lysis, as is seen in this study with respect to the *waaL* mutant. These findings further indicate that perhaps binding of HlyA to the R1 core moieties contributes to serum resistance in CFT073, through stabilizing the outer membrane and the interactions between O6 antigen and the K2 capsule, as depicted in Fig. 6. Interestingly, mutations to the most abundant *E. coli* protein, surface-exposed Lpp (or Braun’s lipoprotein) has been shown to be essential for maximum K2 capsule surface association in CFT073, and so perhaps HlyA plays a similar role in capsule retention (Diao et al. 2017). This hypothesis is supported by the reduced capsule seen in *hlyA* mutants compared to wild-type. It is notable that K2 also relies on interactions with the R1 core to remain associated with the cell, as *waaL* mutants display ~50% less capsule than wild-type, whilst *waaG* mutants only possess 10% of wild-type level capsule (McGarry et al. 2024). It is the amphipathic nature of HlyA, which permits its binding to LPS and Ca^2+^, indicating that for both K2 and HlyA, electrostatic interactions at the cell surface are essential for virulence and serum resistance (Herlax et al. 2005). The proposed model for HlyA in K2 surface retention is depicted in Fig. 6.

## Supplementary Material

xtaf009_Supplemental_Files
