# Supplementary material for: Role for RTX-family toxin HlyA of extraintestinal pathogenic Escherichia coli in serum resistance
Source: FEMS Microbes. 2025 Jul 2;6:xtaf009. doi: 10.1093/femsmc/xtaf009 (PMC12231138; doi:10.1093/femsmc/xtaf009)
Supplement: xtaf009_Supplemental_Files [file xtaf009_supplemental_files.zip › FEMSMC-2025-005.R1 one sentence summary.docx]

A common exotoxin of ExPEC was shown in this study to contribute to serum resistance, potentially through interactions with surface capsule in an ExPEC prototype, CFT073.
