# Supplementary material for: Role for RTX-family toxin HlyA of extraintestinal pathogenic Escherichia coli in serum resistance
Source: FEMS Microbes. 2025 Jul 2;6:xtaf009. doi: 10.1093/femsmc/xtaf009 (PMC12231138; doi:10.1093/femsmc/xtaf009)
Supplement: xtaf009_Supplemental_Files [file xtaf009_supplemental_files.zip › Supplementary file_HlyA.docx]

**Supplementary file**

| **Strain** | **Genotype/ Description** | **Selectable marker** | **Reference** |
| --- | --- | --- | --- |
| ***Escherichia coli*** |  |  |  |
|  |  |  |  |
| CFT073 Wild-type | Prototypic urosepsis isolate; O6:K2:H1 serotype | None | (1, 2) |
| CFT073 Δ*ksl* | *ksl::kan* | Kan^R^ | (3) |
| CFT073 Δ*ksl::FRT* | *ksl::FRT* | None | (4) |
| CFT073 Δ*waaL* | *waaL::kan* | Kan^R^ | (4) |
| CFT073 Δ*waaL::FRT* | *waaL::FRT* | None | (4) |
| CFT073 *ΔhlyA* | *hlyAl::kan* | Kan^R^ | This study |
| CFT073 Δh*lyA::FRT* | *hlyA:FRT* | None | This study |
| CFT073 Δ*waaG* | *waaG::gent* | Gent^R^ | (4) |
| CFT073 Δ*waaG::FRT* | *waaG::FRT* | None | (4) |
| DH5α | K-12, serum sensitive control, *fhuA2 Δ(argF-lacZ)U169 phoA glnV44 Φ80 Δ(lacZ)M15 gyrA96 recA1 relA1 endA1 thi-1 hsdR17* | None | New England Biolabs |
|  |  |  |  |
|  |  |  |  |

**Table S1 Strains used in this study.** Kan - Kanamycin, Gent – Gentamicin.

| **Plasmids** | **Description** | **Resistance** | **Reference** |
| --- | --- | --- | --- |
|  |  |  |  |
| pCP20 | Possesses FLP flip recombinase gene, 30°C temperature-sensitive replication | Amp^R^ | (5) |
| pKD4 | Possesses FRT-flanked kanamycin resistance cassette | Kan^R^ | (6) |
| pKD46 | Used to construct mutants through homologous recombination, possesses λ-Red recombinase genes *exo bet* and *gam* which are induced by L-arabinose, temperature sensitive replication at 30°C | Amp^R^ | (6) |
| pCL1920 | Cloning vector backbone used | Spectinomycin^R^ | (7) |
| pHlyCABD | pCL1920 encoding *hlyCABD* for complementation of *hlyA* mutant | Spectinomycin^R^ | (8) |

**Table S2 Plasmids used in this study. .** Kan - Kanamycin, Gent - Gentamicin, Amp – Ampicillin.

| Name | | | | Sequence (5’-3’) | | Purpose | |
| --- | --- | --- | --- | --- | --- | --- | --- |
| 1 | | ACAGATTTCAATTTTTCATTAACAGG | Amplify hlyA gene | | | |  |
| 2 | | TGACAAGAATCCATTATGACTCC | Amplify hlyA gene | | | |  |
| 3 | | **TTATCT**AGAGGGTACTGGGAAGACCAGGGTTA | Clone HlyCABD (**flank to *KpnI***) | | | |  |
| 4 | | **ATAGGT**ACCTTAACGCTCATGTAAACTTTCTGTT | Clone HlyCABD (**flank to *XbaI***) | | | |  |
| 5 | | TTGCCTTCCAGGCTGTTATC | *rplT* housekeeping control RT-PCR | | | |  |
| 6 | | CTGCTTTCGCTTTTTCAACC | *rplT* housekeeping control RT-PCR | | | |  |
| 7 | | CCCGTCATACTGACTGAGTACAT | *ksl2A* (region 2 capsule gene) RT-PCR | | | |  |
| 8 | | TGCGGTGATTTGCAGTATCC | *ksl2A* (region 2 capsule gene) RT-PCR | | | |  |
| 9 | | CGCAAGCGATCTGTTTACCG | *kpsC* RT-qPCR | | | |  |
| 10 | | TTAAGCCTGGCGCCCATAAA | *kpsC* RT-qPCR | | | |  |
|  | |  | | | |  |  |

**Table S3. Oligonucleotides used in this study.**

**
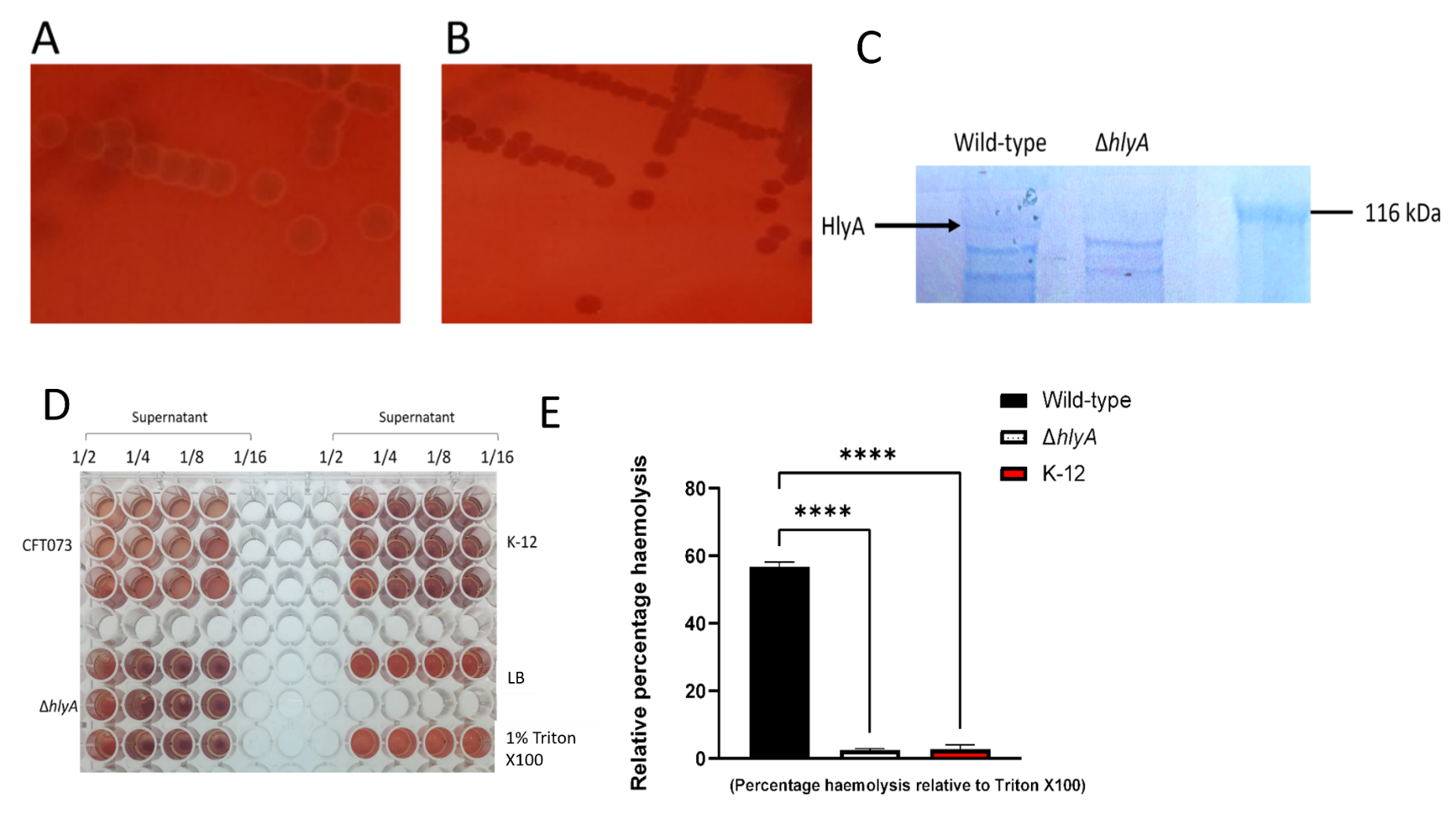
**

**Figure S4** **Mutant lacks Haemolytic activity.** Colonies from LB agar plates were single colony purified onto blood agar, grown overnight at 37°C and examined for haemolysis. Wild-type CFT073 colonies (A) displayed qualitative haemolysis compared to the Δ*hlyA* colonies (B) which did not appear to lyse red blood cells. (C) Supernatants from wild-type and *hlyA*-deficient CFT073 were concentrated 10-fold using Amicon® Ultra Centrifugal Filter devices (50 kDa cutoff – Merck) before separation by SDS-PAGE on precast Novex Tris-Glycine 12% gels. Gels were stained with Coomassie blue to visualise proteins. (D) Wild-type supernatants displayed qualitatively more haemolysis compared to the Δ*hlyA* supernatants, K-12 supernatants and untreated (LB) control, serving as negative controls for haemolysis. Some CFT073 supernatants lysed RBCs to levels comparable to the detergent Triton X100, a positive control for the lysis of RBCs. Relative percentage haemolysis was calculated as OD_540nm_/OD_540nm_ Triton (x100) and displayed in a bar chart (E). Statistical significance calculated by One-way ANOVA and Tukey’s and is shown relative to wild-type haemolysis. N=3 biological replicates


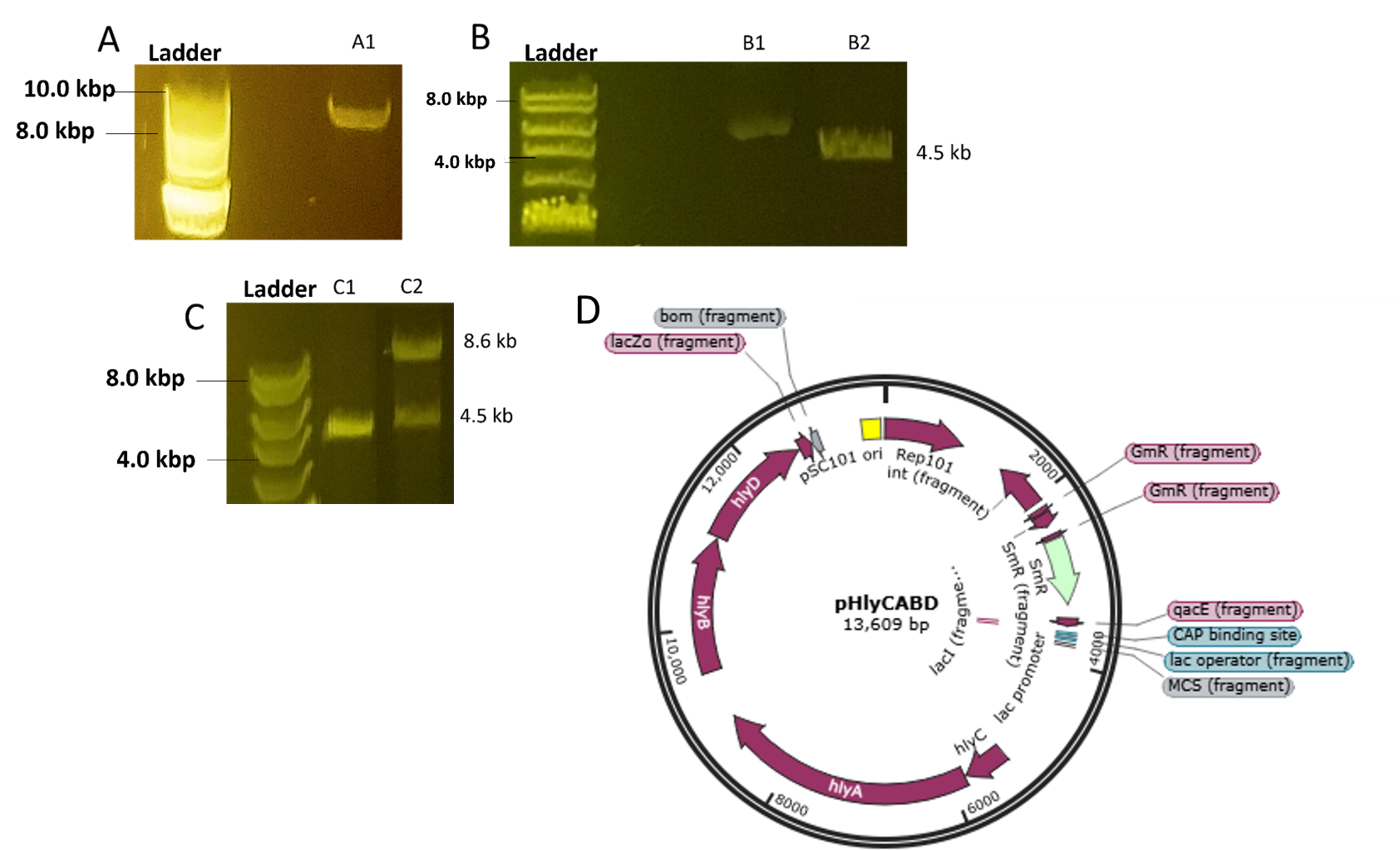


**S5. Cloning of *hlyCABD*.** A) *hlyCABD* was amplified (A1) with primers containing *Xba*I and *Kpn*I sequences. In (B) the amplicon from (A) was digested with *Xba*I*/Kpn*I (B1), as was pCL1920 (B2). Following ligation and transformation, clones were screened in (C). C1; pCL1920 empty vector was digested with *Xba*I*/Kpn*I. C2 is putative clone, also digested with *Xba*I and *Kpn*I*.* C2 was sequenced due to the correct size of digested products. (D) resulting sequenced vector, showing correct orientation.

**References**

1. Welch RA, Burland V, Plunkett G, Redford P, Roesch P, Rasko D, et al. Extensive mosaic structure revealed by the complete genome sequence of uropathogenic Escherichia coli. Proc Natl Acad Sci U S A. 2002;99(26):17020-4.

2. Guyer DM, Kao JS, Mobley HL. Genomic analysis of a pathogenicity island in uropathogenic Escherichia coli CFT073: distribution of homologous sequences among isolates from patients with pyelonephritis, cystitis, and Catheter-associated bacteriuria and from fecal samples. Infect Immun. 1998;66(9):4411-7.

3. Miajlovic H, Cooke NM, Moran GP, Rogers TR, Smith SG. Response of extraintestinal pathogenic Escherichia coli to human serum reveals a protective role for Rcs-regulated exopolysaccharide colanic acid. Infect Immun. 2014;82(1):298-305.

4. McGarry N, Roe D, Smith SGJ. Synergy between Group 2 capsules and lipopolysaccharide underpins serum resistance in extra-intestinal pathogenic. Microbiology (Reading). 2024;170(8).

5. Cherepanov PP, Wackernagel W. Gene disruption in Escherichia coli: TcR and KmR cassettes with the option of Flp-catalyzed excision of the antibiotic-resistance determinant. Gene. 1995;158(1):9-14.

6. Datsenko KA, Wanner BL. One-step inactivation of chromosomal genes in Escherichia coli K-12 using PCR products. Proc Natl Acad Sci U S A. 2000;97(12):6640-5.

7. Lerner CG, Inouye M. Low copy number plasmids for regulated low-level expression of cloned genes in Escherichia coli with blue/white insert screening capability. Nucleic Acids Res. 1990;18(15):4631.

8. Buckles EL, Wang X, Lane MC, Lockatell CV, Johnson DE, Rasko DA, et al. Role of the K2 capsule in Escherichia coli urinary tract infection and serum resistance. J Infect Dis. 2009;199(11):1689-97.
